# Clinical Cases, with Remarks

**Published:** 1893-03

**Authors:** Edward Mahony

**Affiliations:** Liverpool, Eng.


### CLINICAL CASES, WITH REMARKS.

EDWARD MAHONY, M. D., LIVERPOOL, ENG.

1. Miss D., aged thirty-four, March 17th, 1891, presents herself with the following symptoms:

*Moral.* Takes things quietly; has had trouble.

*Head.* Once in six weeks severe headache; better keeping quiet.

*Heart.* Palpitation on ascending; no murmur.

*Back.* Easier with something pressed against it.

*Sexual.* Menses since thirteen, always excessive, with aching in back, especially lower part. Blood sometimes too dark. Lasts eight days; only fifteen days between each period. *Before,* feels good for nothing. *During,* sleepless first night, must support back with pillow. Hot turns lately followed by perspiration; faint, with sinking at epigastrium toward supper time. *After* menses feels quite well.

*Stool.* Costive since influenza, twelve months ago.

*Skin.* Easily perspires.

*General.* Worse in dull, foggy weather or spring. Used to be very thin, now plump. Had rheumatic fever following scarlet fever at the age of eight. Calc-c.$^{200}$, one dose.

March 25th.—Feels brighter, back improved, also longer intervals between faints. Constipation decidedly better.

April 3d.—Severe headache day of last visit. Back greatly improved. Feels generally brighter. No symptoms of menses.

April 17th.—"Feels much better, much better." (Own words.) No headache since. Has had menses, with decidedly less pain in back.

May 2d.—Recurrence of menses in fourteen days, no pain in back, profuse, one week; not the usual weakness.

June 9th.—Pain slightly in back three times. One period at an interval of seventeen days, with very little pain. Some return of constipation, with desire for stool. No headache since. Feels stronger and better in herself. Nux-v.$^{200}$, one dose.

July 3d.—The above (Nux-v.) acted for two or three days. Return since of constipation, with, however, absence of desire. The bowels have been irregular since the drugging for influenza a year ago. She has now pricking in anus as of needles and pins and much itching at anus. Calc-carb.$^{500}$, one dose.

July 14th.—Less constipation; pricking still or soreness after stool and itching of anus at night.

*Menses* profuse, prolonged three days, but not much pain; back wonderfully better. Felt weak for a short time; period only just over.

*Remarks.*—This patient received in all three doses of medicine, two of Calc. of different potencies and one of Nux for the symptoms mentioned. She has not been to see me since, but I have heard of her from immediate relatives, and know that, in addition to her required work, which is in the Post Office, she has had a most prolonged strain in helping to nurse her poor mother, who came to me for a short time with a fungoid wart in the rectum, but suffering at times much pain, and friends (?), of course, endlessly worrying her to have something done, she put herself under a practitioner of the old school, who performed endless operations, apparently cauterizing in some way, and she has suffered at least as much from this as from the original disease.

I mention the circumstance as indicating probably further psoric or probably sycotic taint in the daughter (hereditary), and consequently the exceedingly satisfactory action of Calc., especially with the influenza drugging in addition.

2. Mrs. F., aged thirty-seven. The mother of six children. Had and has much hard work. Energetic, somewhat anxious temperament. Small; used to be stout, now somewhat thin.

Came to me May 7th, 1891. Haziness over eyes worse from reading or sewing.

One dose of Nat-mur.$^{30}$, night and morning, for eight days, removed this, but she reappeared, December 23d, with the following symptoms:

*Chest.* Inward coldness working to the left and then gradually all over; occurring in paroxysms of two hours; feels faint with it; comes on twice a day; oppression at night.

*Sexual.* Menses absent since October.

*Stomach.* Pains nights, palliated by spirits; these she never takes except medicinally.

*Flatulence.* Pastry and potatoes disagree.

*Stool.* Constipation without desire.

*Sleep.* Lies awake, on right side or back. Dreams of work. Drowsiness at 2 P. M.

*General.* Must have everything loose. Has taken Puls. for three weeks until a fortnight ago; this would be a low potency. On auscultation the heart-beat was rapid, with a clicking sound.

*Extremities.* Cold feet.

Considering the inward coldness and drowsiness at two o'clock in the afternoon were probably results of the Puls., and her temperament suiting it likewise, she received one dose of Nux-v.$^{30}$.

January 1st, 1892.—Very much better, but still has afternoon drowsiness, constipation, and cold feet; also as new symptoms:

*Extremities.* Pain and coldness in the left arm.

*Fever.* Thirst for large quantities; tongue dry and hard; worse toward evening. Lycopod.$^{200}$, one dose.

January 18th. All symptoms relieved except the drowsiness.

February 6th.—Complains of nothing, and on auscultation the cardiac sounds are normal.

*Remarks.*—In this case, again, one dose of each indicated medicine proved sufficient, first, to antidote the overdosing with Puls., and next to remove the symptoms (suppressed ?) that then appeared. I should like here to indorse the experience of other colleagues as to the value of Nux and Puls., whether to antidote each other or previous allopathic prescribing; the distinction between them is so easily made in the moral sphere that there is no excuse for anybody not being able to make it, and for myself I may say that almost universally I give but one dose, usually 200, and the way it clears the ground and enables the true symptoms to develop is highly satisfactory. One can promise the patient improvement by the next interview.

3. Rennie G. Eleven and a half months.

January 9th, 1891.—After the administration of Corall-rubr.$^{200}$ for pertussis with red or black face, father reports: Whoops only once or twice, and that about evening. Difficulty in getting to sleep at night. Very fretful, and often coughs without whooping. Navel protrudes; has always been a rare screamer. (N. B.—The father is stone deaf since scarlet fever when five years old, so this is probably the mother's expression.) Inside tops of thighs red-raw for a long time. Points of teeth can be felt. Bell.$^{30}$.

February 12th.—Stools at times loose and most offensive. Soreness inside thighs better. Sil.$^{30}$.

March 6th.—"The medicine sent seemed to act like magic; for, next morning, he seemed quite like a new little man, and in a few days was fairer and fatter than ever. Now coughs violently and for long periods, with a sound resembling sneezing (the father, we must remember, is stone deaf), choking, exhausting. Has had most offensive vomit and stools, treated by Sulphocarbolate of Soda." Agaric$^{30}$.

April 16th.—"So fat and rosy he seems none the worse for late illness."

*Remarks.*—This case was treated, the family living about eighty-seven miles away, entirely by correspondence; hence there is not the careful note what symptoms are relieved or gone that one could wish. Still the Bell. had probably removed the fretfulness, screaming, and difficulty in getting to sleep, or some reference would have been made to these.

The remarks on the action of Sil. are highly satisfactory, and make it the more regrettable that Sulpho-carbolate of Soda was interposed in the way it was.

As to correspondence cases, a difficulty I suppose most find is that patients will not, many of them, take the trouble to say how the different symptoms are as compared with previous reports. The public do not seem educated yet to this point.

4. August 11th, 1891. W. H., æt. forty. A miner. Not able to do much the last four years. A medium height, spare man, with reddish hair and whiskers. "Done feeling at times in the belly." Flushes of heat to face accompany his symptoms generally. Burning and heated sensations. Heaviness of head. Fullness after meals. Costiveness with alternations of loose stools, without desire. Potatoes and meat disagree. Weak in loins mornings. Frequent waking. Urine at times copious and light-colored. Worse in himself in damp weather. Liable to faints. Was under true Homœopathy four years ago with benefit.

*On examination* nothing abnormal in abdomen. Slight cardiac murmur. Sul.$^{200}$, one dose.

25th.—After aggravation, improvement.

September 5th.—Had good-for-nothing feeling pass over him which seemed to spring from the heart. Is better in bed and when quiet; when moving about feels a kind of swaying in his body. The right leg is painful at times, weakness in both knees, aggravated by movement. Natr-mur.$^{1000}$, one dose.

18th.—Better, hoarseness same night; heart greatly relieved, return of pain in right side, also eye symptoms (these had not been reported to me before). Small piles with, at times, severe itching; has had them larger with bleeding, causing great weakness.

October 10th.—Much better, heart not having plagued him much during the three weeks. Piles larger and more unpleasant for a day or two, but now smaller and easier, except at night on going to bed, when the itching is troublesome.

15th.—Since a wetting of feet and legs, pain in ankles and legs, and has had heart upset also twice. Two powders each, eight doses. Sil.$^{30}$.

November 3d.—Took one powder only. Good-for-nothing feeling again and again.

12th.—Felt a great deal better. A great deal more energy since the 8th especially.

26th.—Feeling on the whole better, save good-for-nothing feeling now and again, though not like they used to be. Cold across loins. Sil.$^{30}$, as above.

December 29th.—"Return of those nasty feelings." Inclination to weep. Sil.$^{50m}$, three doses.

January 22d, 1891.—Attacks are accompanied by passage of much colorless water. Lips sore and blistered. Sulph.$^{50m}$, one dose.

March 5th.—Much better. Since catarrh two days ago pain across loins, tickling cough. Bry.$^{cm}$, one dose.

*Remarks.*—In this case the action of the one dose of Sulph.$^{200}$ is distinct; also of Nat-mur.$^{1000}$—hoarseness same night, heart greatly relieved, and then return of several old symptoms. The Sil. given in the 30th the second time would probably have been better in one dose higher, yet the three doses of 50m seem to have caused no bad symptoms. The last dose of Bry. was given in accordance with the remarks in Vol. I, *Chronic Diseases*, on page 166, on some of the accidents by which the cure of chronic diseases may be temporarily disturbed.

5. Mrs. H., æt. fifty-one; no children.

May 7th, 1891.—*Moral* and *head.* Low-spirited. Pain left occiput after food.

*Face.* Florid, swelling of forehead.

*Gastric.* Craving at night, satisfied by very little. In daytime little satisfies.

*Abdomen.* Pain left side as from incarcerated flatulence.

*Stool.* Costive.

*Sexual.* Menses, scanty, many years, pain most at bottom of stomach.

*Extremities.* Cramps at nights when warm in bed, feet burn.

*Sleep.* Sleepless, frequent waking.

*General.* Worse in morning and from damp.

Had fever and ague twenty years ago in New York.

Is tall, stout, flabby. Sulph.$^{200}$, one dose.

14th.—Return of old trembling in the left side. Head improved, abdomen, limbs, sleep, all better and stomach symptoms.

26th.—Return of old pain in back; trembling still. Sleep not quite so good. Sulph.$^{400}$, one dose.

11th.—Pain across loins most on left side, with thick urine (an old symptom not felt lately). Return of occipital pain also; sleeps better, but rambles a good deal; wakes unrefreshed. Burning in soles of feet.

Considers herself almost well, had placebo, and has not returned since.

There seems nothing to remark here, but the unvarying tale that if antipsorics are administered in this style, they cure, cause temporary aggravations, and temporary return of old symptoms.

6. February 25th, 1891.—Miss J. Sores in mouth treated by washes. Acidity. Aversion to meat. Desire for milk. During the last twelve months has used lotions occasionally to mouth. Takes much salt. Temperament nervous, much mental work, being one of the heads of a large scholastic establishment. Nux-v.$^{200}$, one dose.

March 18th.—Improved in all ways. An attack of diarrhœa a week ago lasting some days.

Is worse in herself in damp weather. Trifles disturb. Calc-c.$^{200}$, one dose.

July 15th.—Wakefulness early night, constitutionally, aggravated now from over-mental strain. Sep.$^{30}$.

November 5th.—The above relieved the wakefulness in a short time. Now, heaviness everywhere, with stiffness in joints generally. No general symptoms, but purposes a visit to Buxton for the mineral waters. A few doses of Nux-v.$^{30}$, followed by Calc-c.$^{30}$, however, enabled her to continue her duties and in a few days she was restored to health without the inconvenience and expense of the proposed change to Buxton. This case again illustrates the power of one dose of Nux-v. to change the system after twelve months of local applications, and its action was further proved by the diarrhœa of some days followed by improvement in all ways.

7.—Miss J.

August 5th, 1891.—Has had influenza three times, last time in July, treated allopathically. Present symptoms:

*Stomach.* Tongue coated, anorexia.

*Stool.* Two or three per day, loose.

*Averse* to sugar, meat. *Likes* salt always.

*Sexual.* Menses too frequent, copious, pain left ovarian region.

*Skin.* Perspires much.

*Sleep.* Dreamy, unrefreshing.

*General.* Worse in damp, hot weather, better in frost, or chilly air. Has had Quinine. Natr-mur.$^{200}$.

6th.—Same night, violent aggravation, neuralgia one hour, (trigeminal), then suddenly ceased.

20th.—Distinct improvement. She writes, "I got quite a start for the better after the reaction of the first dose passed off. I find it difficult to distinguish between the effects of the medicine and my own symptoms. * * * I had a turn of feverishness and shivering which lasted more than twenty-four hours and passed off with a perspiration. The shiverings were so much as to wake me out of my sleep, and I took a weak dose of Acon. which relieved me. That is the only time I have had recourse to any other remedy. My appetite is decidedly better. * * * I am sleeping better, but dream tremendously, generally such possible dreams that I hardly know if the things have happened or not, perhaps only know I have slept because I have dreamt, and get up unrefreshed and tired with the night's experiences. For the last week I have had a noise in my ears like the whirr of machinery. I did not notice it at first when lying down but now have it night and day. Frequent frontal headache and my eyes feel hot under the lids; my hair falls out very much and my back and legs get very tired and stiff; often have a pain in my back under the shoulders. A little exertion makes me very tired and I seem to get a slight cold and then this pain and stiffness from the waist all down to the calves of the legs, but all this is only what I had for three or four months after the influenza last year." All this, it is to be observed, was the result of two globules per night of Nat-m.$^{200}$, from the 5th to 20th of August. I then sent placebo and the next report is:

September 18th.—Aggravation continued so much that she stopped the medicine (placebo). Menses less profuse and somewhat less painful the first day, but was very poorly afterward, and exhausted; gets, also, neuralgic headache now, and hands are sometimes very burning.

Continue placebo.

October 24th.—"Felt so well did not write sooner." After a while stiffness went and indigestion almost, and in every way has been very much better. A little return of indigestion since last week, but nothing fresh. Had a fall which caused some pain, especially in left side, on which she fell. Continuous headache, since 21st, in forehead; also, the last few days, occasionally burning in hands, but much less often since last time of writing. Nat-mur.$^{1000}$, one dose.

January 16th, 1892.—Reports: From piercing wind, roughness, and pricking in throat, then violent cold in head, sneezing, etc., most of evening. Cough wheezy. Used to be very subject to this kind of thing. Has taken Acon. and Merc. States that left lung used to be tender and susceptible; likes salt, dislikes meat and sugar. Phosph.$^{200m}$.

I did not hear again, but early in February she brought a friend to see me, and took the opportunity of saying that she was now quite well.

*Remarks.*—The aggravation sharp and short, the same night after two globules Nat-m.$^{200}$ is interesting, as also the prolonged aggravation from several doses. Had she been within reach she should not have had the opportunity of this, but, not being able to see her after the first time, I ventured the repetitions. I do strongly feel that, considering how we are situated with regard to many of our patients, the question of repetition is a most difficult one. I feel much inclined to indorse the opinion of the late Dr. R. R. Gregg, that it is *the* most difficult question in Homœopathy.

F. L. A young servant girl of about eighteen, whom I had previously cured of two nasal polypi which used completely to block the nostrils when swollen with catarrh.

She wrote, on January 26th, 1891, with, among others, the following symptoms : Can't lie straight in bed, the bed not seeming strong enough to hold her, but gets better after a time. Headache, mostly on the temples, and on stooping ever so little a terrible pain on the top ; also on going up-stairs. Swelling of the ankles on rising in the morning, making it very painful to walk, especially to go down-stairs. "I seem unable to bend them, the legs the same all the way up, and I don't quite lose it all day. My knees, too, are so sore, causing pain on use. Altogether, sir, I feel regularly done up, and would gladly be quiet, not to see or speak to any one. When I do sit down, it's the greatest hardship to move again, and I am so irritable, scarce knowing how to bear myself." Sil.$^{200}$, one dose.

*Remarks.*—This one dose cured, and I have not been applied to since. This case is another confirmation of the Sil. symptom, swelling of the ankles in the morning, and on that account seems worth recording. The peculiar mental symptom of the bed not seeming strong enough to hold her I am not acquainted with, but Canth. has sensation as if would fall through the bed, which seems very similar.

M. E., a cook, at the Climacteric.

January 9th, 1891.—Nausea from all food and continuously. Pain through left chest backward occasionally. Palpitation severe at times. Headache. Menses absent six weeks. Ipecac$^{200}$.

12th.—Better in all ways. Nausea went in twenty-four hours. Return of an old pain in the left side of the back. Better from rubbing. Also worse in bed. Return of heart throbbing. This throbbing had occurred previously on October 13th, 1890, and is thus recorded : Heart at times throbs very quickly, and all the body in unison with it, generally in the afternoon, and lasting about three hours. She then received Tellurium$^{6}$, three doses, one night and morning, and reported on November

5th : The throbbing does not come on so often. A fortnight ago hysteria and faintness came with something else (period), but in a day or two it passed off, leaving some pain and inconvenience the remainder of the week, but less than usual. Bearing this in mind, she now got Tellurium$^{6}$, eight doses, and reported in a week. Sickness and pain inside gone, appetite better. Lower back very weak, difficult to rise from a stooping position. Calc-c.$^{200}$, one dose.

January 30th.—"My usual health is again restored."

March 12th.—Return of nausea and headache, though not so severe. Has tried Ipec. (potency not stated, but probably low) without benefit. Ipec.$^{1m}$, F. C., one dose.

No further treatment was required until after a cold she reported, June 21st, rawness on breathing; cough, restless nights. Sulph.$^{200}$, eight doses.

July 2d.—Chest much relieved; weakness terrible. Nil.

October 7th.—Again from cold, shooting pains in malar bones and chest. Sul.$^{30}$, for one week.

13th.—Cold wonderfully relieved, but strength greatly reduced. Calc.$^{30}$, one dose.

27th.—Much stronger. Pain from heel right up back of legs; heat, especially right side. Puls.$^{200}$, eight doses.

November 28th.—After amenorrhœa seven months, had a return for two days, followed by vomiting, with pain from lower spine to top of head. Lach.$^{30}$, eight doses.

30th.—Menses returned yesterday.

December 8th.—Only trouble now, weakness. Joints all feel loose. Stram.$^{200}$, eight doses.

Nothing further was required until the 3d of the present month, March, when she reported: Following a stiff neck on the right side, a sore spot immediately above the collar bone, very sore to the touch, and on drawing a long breath causes pain. Underneath right shoulder-blade a pain, this last is new to her. Chelidon$^{200}$, one week.

March 8th.—Neck nearly all right, soreness too; previously had no power to lift head from pillow. Only took one of the powders. Pain under right shoulder-blade better. Yesterday and to-day (7th) felt sickly with headache. On the ground of its family relationship, a few doses of Nux-v.$^{200}$ were sent and she was soon all right.

*Remarks.*—My principal reason for reporting this case is its confirmation of the characteristic Tellurium symptom, Palpitation of the heart and general throbbing through the whole body with fullness of the pulse. Tellurium also has caused premature menses. The weakness induced also by Sulph. when repeated though in such different potencies, 30 and 200, is interesting, especially as there was no appreciable aggravation of symptoms from other medicines.

H. I. M., æt. seventy-seven. This gentleman is a good illustration of Anglo-Indian life as found among Europeans, having returned to this country after some thirty years in India, where he had decided sunstroke, and got his system saturated with salt, having been engaged in government salt works. This eventuated in thorough depression of the nervous system and complete loss of sexual power, after having been the father of five or six children. He got considerable relief from treatment at my hands, including return of sexual desire, which had been absent many years, and again applied to me.

April 13th, 1891.—Stating that he had been under a local homœopath in the town in which he resides for bronchitis, and made a good recovery, but was now suffering from confusion of mind from reading, with depression of spirits. Coccul.$^{30}$, twice a week, eight powders.

28th.—Aggravation followed by relief. Nil.

May 28th.—Quiet, steady accession of strength and flesh. Confusion of brain still with forgetfulness of names. Puls.$^{200}$, eight powders, one twice a week.

June 17th.—" In almost every respect in the best of health." Mental depression from loneliness. Aur.$^{30}$, four powders, one per week.

July 4th.—After three powders all traces of the long-continued painful depression have quite disappeared ; in fact, he feels in all respects well. Memory going especially for names. Four powders of Lycopod.$^{30}$, one per week.

August 27th.—Dread of mind giving way, from being lonely, spiritually, and general aggrieved feelings. Sullen mood. Feeling of being abandoned. At times foolishly merry (an old symptom). Low voice, indistinct, worse on raising his voice, which often causes tremor of speech. Carb-anim.$^{30}$, one per week, four powders.

September 26th.—Depression gone. Memory flighty and fluctuates, for names especially. Frequent huskiness of voice, with, at times, difficulty, of audible expression. Aggravation from hot weather. Graph.$^{2000}$, one dose.

November 14th.—Memory improved, though having had much domestic worry. Has "an active eruption of scabies on both legs below knees." Graph. rep., one dose.

December 10th.—Much domestic worry leaving weakness. Eight powders, Staph.$^{200}$, one twice a week.

January 25th, 1892.—Again complains of loss of memory. Eight powders, Conium$^{200}$.

February 10th.—Powders were acting favorably on his memory when he got lumbago from a draught of air; took Bry. and Nux-v.

*Remarks.*—It will be observed that in this case the symptoms prescribed for were purely mental and moral. This was because other symptoms were not reported, and shows how much can be done even in very imperfectly reported cases, and in the aged with evidently failing vital powers if only something like characteristic symptoms can be found. The action of the Aur. is instructive and still more the return of the scabies eruption, his own words, after the one dose of Graphites.

Mrs. W——; widow; two children; æt. forty-one.

This lady is of Jewish extraction. Her mother is living, at the age of eighty-two, and hearty in many ways. One brother, of neurotic temperament. She, herself, highly nervous. Her husband was ordered to New Zealand for consumption, whither she accompanied him. He died there, and she, shortly afterward, was put in an asylum; an action she resents even now in the strongest way as unjust. She was, on recovery, said to be consumptive, and ordered back to this country on that account. She consulted me.

March 10th, 1890.—On auscultation, slight dullness both apices anteriorally, and at left apex where it was said the mischief was, was roughness on inspiration and expiration. Pain at left apex comes and goes suddenly ; worse from touch. Hot feeling there. *Face,* flushes of heat at times.

*Stomach.* Sinking at epigastrium eleven A. M. six months. Rush of blood to head.

*Moral.* Irritable; nervous. Neither now nor at any time since have there been delusions or signs of mental defect.

*Sexual.* At the end of last November, menorrhagia, lasting three months, with copious menses at the usual time. Sulph.$^{200}$, one dose.

21st.—Appetite greatly increased. Inward bleeding piles yesterday, relieving the head, and pain in left foot. This last she has not had for some years. Looks and feels better. Sinking at epigastrium has been worse. Pain in left chest is worse. Nil.

April 21st.—Slight roughness of respiration at affected spot. Sinking at epigastrium less; pain in left foot gone. *Menses* appeared March 23d ; copious ; she was weak toward end. Sulph.$^{4000}$, one dose.

May 12th.—For two or three days increased irritable feeling with inability to think, also trembling of left arm and one leg. She now informs me that before and since her last confinement, about four years, she has had varicose veins on the left side, and itching of feet. Perspires most days. Is worse in damp weather. Has lost one stone in three weeks. Used to be plump. Calc-c.$^{200}$, one dose.

June 2d.—Felt almost well until five days ago, when great prostration came on until yesterday evening; better to-day. Warm things relieve the head.

Return of an old pain in the chest the day before yesterday and phlegm at top of throat. No medicine.

June 23d.—Return occasionally of an old facial neuralgia. Pain in right chest similar to what she has had in the left chest. Weaker. Has gained flesh. Calc.$^{200}$, three doses.

July 14th.—Did not begin the medicine for a weak. Menses were on at the time of her visit to me, and lasted a fortnight. No medicine.

August 11th.—Languor at times. Menses, followed by colored matter, with itching (new).

Gnawing and clutching at epigastrium before meals. Acid-phosph.$^{200}$, one dose.

September 8th.—Had a great sinking at epigastrium the evening of the day on which she had the Acid-phosph. Languor ceased. Return more severely of pain at left apex, also a pain in back. Bleeding hemorrhoids day before yesterday. Menses copious and weakening, but no itching. Sinking at epigastrium, better from food. Sulph.$^{50m}$, one dose.

October 2d.—Appetite good. Does not feel languor and sinking at stomach; has had neuralgia. Occasional severe pains at chest. Before menses, last week unusual pain in back and neck, blood-vessels of latter seemed almost bursting; heart thumping, one evening, sounded to her like the loud ticking of a clock; greatly relieved when menses came on (all this new). For a few days was most unusually ill. Iodium$^{200}$, one dose.

November 3d.—Sound like bellows in the left ear with palpitation. Great pain at upper back. Pain in chest. Nervous dread of horses since being frightened by them nearly four years ago. Pain at angle of right scapula. Small, reddish patch left side of tongue. Hairs turn gray. Chelid.$^{200}$, eight doses.

20th.—Pain in back gone in a few days. Feels very much better. Chest pains gone. Feels a nervous restlessness; must be doing something; long in getting to sleep at night; brain too active at the wrong time. Night before last a spasmodic pain in the left side of the head, for an instant, similar to what she once had in New Zealand. Nose bled twice two or three days after seeing me. Hairs turn gray. Fright from horses at four years of age. Lycopod.$^{200}$, one dose.

January 27th, 1891.—After disagreeable interview with a friend return of pains. Staph.$^{200}$, eight doses.

February 5th.—After medicine pains in knee, then thigh, extending to hip; now gone from these and in neck, extending on movement to right shoulder-blade. Anorexia. Great weakness. Constipation, with strange sensation in head; took salts which did no good. Frightened at the constipation, as this preceded the previous illness in New Zealand. Puls.$^{200}$, one dose.

August 29th.—Day before yesterday upset from returning to old associations, relieved by a good cry. At times, after worry, bleeding piles. Menses every five weeks, copious. Neuralgia still in right side of face, relieved by reading or other mental work, which causes drowsiness. Pain at one spot on forehead. Ign.$^{200}$, one dose.

*Remarks.*—This case is interesting in several ways: 1. There is no family history on either side, that I can discover, of consumption; hence, the question naturally occurs, did her husband infect her? There is nothing else to put down as the cause of the apparently commencing tuberculization, as her surroundings immediately preceding its onset were, on her husband's account, such as would tend to throw off, rather than induce, such a condition. 2. Her husband having phthisis, is ordered to New Zealand, and dies—she, having the commencing symptoms of it is, on this account, ordered to England and lives. She has, too, been through the unusually severe weather that has marked the last two winters, and the preceding spring also, and thriven; has been, too, within forty miles of Liverpool the whole time and *not* in the milder climate that, according to theory, such patients should have. 3. The treatment has, as will be noticed, entirely ignored names of disease and pathological indications, and each medicine was given because symptoms pointing to that medicine were prominent. This, as should be, according to the law, while benefiting caused return of old and, no doubt, suppressed symptoms as the pain in the left foot after one dose of Sul.$^{200}$, pain in chest after one dose of Calc.$^{200}$, also neuralgia, facial. The prompt aggravation after one dose of Acid-phosph., followed by removal of languor and return of left apex pain is also instructive. She has been practically well, doing fair work, both mental and physical, and has needed no treatment since the last entry, August 29th, 1891, and I heard of her only yesterday (March 31st), through her brother, that she considers herself, and is considered by her friends, well. I regret not having examined her chest again since the improvement noted, but the last several consultations were by correspondence.

Mrs. S——, aged about fifty-five. Consulted me last June for early waking with active thoughts. Restlessness, slight noises disturb. Has taken Mercurius with benefit, but the sleeplessness continues. Urine thick at times. Tongue, ashy posteriorly. Calm temperament. Opium$^{30}$, eight doses, one each night.

The one prescription cured.

*Remarks.*—This is a short case—but the prompt action of the Opium given on the two-fold indication of disturbance from slight noises and a calm temperament was in every sense satisfactory. I have known this patient many years and treated her at intervals for various little troubles, but calmness is the index of her mental condition, and, according to Lee's repertory, there is only one other medicine, namely, Veratr-a. having this calmness characteristically, and it is easily distinguished by other symptoms.

